# *In-vitro* antimicrobial, antibiofilm, cytotoxic, antifeedant and larvicidal properties of novel quinone isolated from *Aegle marmelos* (Linn.) Correa

**DOI:** 10.1186/s12941-014-0048-y

**Published:** 2014-10-30

**Authors:** Thankappan Sarasam Rejiniemon, Mariadhas Valan Arasu, Veeramuthu Duraipandiyan, Karuppiah Ponmurugan, Naif Abdullah Al-Dhabi, Selvaraj Arokiyaraj, Paul Agastian, Ki Choon Choi

**Affiliations:** Department of Botany and Biotechnology, AJ College of Science and Technology, Thonnakal, Trivandrum, India; Department of Botany and Microbiology, Addiriyah Chair for Environmental Studies, College of Science, King Saud University, Post box 2455, Riyadh, 11451 Saudi Arabia; Institute of Green Bio Science & Technology, Seoul National University, Seoul, Republic of Korea; Department of Plant Biology and Biotechnology, Loyola College, Chennai, 600 034 Tamil Nadu India; Grassland and forage division, National Institute of Animal Science, RDA, Seonghwan-Eup, Cheonan-Si, Chungnam 330-801 Republic of Korea

**Keywords:** Phenanthrenequinone, Antibacterial, Antifungal, Antibiofilm, Cytotoxic, Antifeedant

## Abstract

**Background:**

Plant metabolites have wide applications and have the potential to cure different diseases caused by microorganisms. The aim of the study was to evaluate the antimicrobial, antibiofilm, cytotoxic, antifeedant and larvicidal properties of novel quinine isolated from *Aegle marmelos* (Linn.) Correa.

**Methods:**

A compound was obtained by eluting the crude extract, using varying concentrations of the solvents by the chromatographic purification. Broth micro dilution method was used to assess the antimicrobial activity and anticancer study was evaluated using MTT assay. Larvicidal activity was studied using leaf disc no-choice method.

**Results:**

Based on the IR, ^13^C NMR and ^1^H NMR spectral data, the compounds were identified as quinone related antibiotic. It exhibited significant activity against Gram positive and Gram negative bacteria. The lowest Minimum Inhibitory Concentration (MIC) of the compound against *Bacillus subtilis* and *Staphylococcus aureus* was 100 and 75 μg mL^−1^ respectively. Against *Escherichia coli* and *Pseudomonas aeruginosa* it exhibited MIC value of 25 μg mL^−1^. The MIC of the compound against *Aspergillus niger*, *A. clavatus*, *Penicillium roqueforti* was 20 μg mL^−1^ and that against *Fusarium oxysporum* (20 μg mL^−1^)*, A. oryzae* (40 μg mL^−1^)*,* and *Candida albicans* (60 μg mL^−1^)*,* respectively. It showed effective antibiofilm activity against *E. coli*, *S. typhii* and *P. aeroginosa* at 8 μg mL^−1^ and did not exhibit considerable cytotoxic activity against Vero and HEP2 cell lines. Additionally, the compound documented significant antifeedant and larvicidal activities against *Helicoverpa armigera* and *Spodoptera litura* at 125, 250, 500 and 1000 ppm concentrations.

**Conclusion:**

The results concluded that the compound can be evaluated further in industrial applications and also an agent to prepare botanical new pesticide formulations.

## Background

In the past 20 years, there has been a renewing interest in identification and characterization of novel metabolites from medicinal plants. These metabolites are mainly used as antibiotics in the treatment of diseases and other pharmacological applications. Due to the rapid rate of plant species extinction, many researchers renewed interest in collection of unidentified plants and its traditional applications, which reduces the time left to explore the decreasing resources [1]. Therefore, urgent efforts are necessary to collect and screen plants in order to determine if they should be conserved for future beneficial use of human kind. Medicinal plants are used by billions of people in most developing countries because of their frequently inadequate provision of modern medicine, their low cost, effectiveness, as well as cultural beliefs and preferences [2]. Treatment of infectious diseases resulted in the development of excessive use of antibiotics which resulted in the antibiotic resistant microbial strains which increase the chance of treatment failure, thus necessitating the search for novel and potent therapeutic agents to treat bacterial and fungal infections [3]. Plants have wide amounts of metabolites that could be used to prevent microbial infections. World Health Organization (WHO) estimates that more than 80% of health care needs in developing countries are met through traditional health care practices [4].

Amongst plants of the *Rutaceae* family, *Aegle marmelos* (Linn.) Correa, widely spread in India, Srilanka, Burma, Thailand and China. The deciduous, alternate leaves, borne singly or in 2′s or 3′s, are composed of 3 to 5 oval, pointed, shallowly toothed leaflets, 4 to 10 cm long, 2 to 5 cm wide, the terminal one with a long petiole. Mature leaves emit a disagreeable odor when bruised. Fragrant flowers, in clusters of 4 to 7 along the young branch-lets, have fleshy petals, green outside, yellowish inside, and 50 or more greenish-yellow stamens [5]. All the parts were used as a traditional oriental medicine for centuries due to several potential effects on the treatment of cough, cold, sore throats, tonsillitis, bronchitis, chest congestion, intermittent fever, intestinal ailments, fertility control and treatment after child birth and fish poison. The primary active constituent such as coumarin, alkaloid, tannin and carotenoid with the number of biological activities has been reported [6]. Previous studies have shown that the identified phytochemicals have antidiabetic, antiulcer, anti-inflammatory, antioxidant, antimalarial, anticancer, antihyperlipidaemic and antiviral activities [7,8]. The aim of the present study was the identification of novel metabolites from *Aegle marmelos* (Linn.) and its antimicrobial, antibiofilm, cytotoxic and antifeedant activities.

## Methods and materials

### Collection of plant

Fresh leaves of *Aegle marmelos* (Linn.) Correa were collected from Kanyakumari district and washed with tap water, rinsed with distilled water, and shade dried for phytochemical analysis and compound extraction. The plant was identified and authenticated with the accession number DBJ-243 in Department of Botany and Biotechnology, AJ College of Science and Technology, Thonnakal, Trivandrum.

### Extraction and purification

The shade dried plant powder (500 g) was placed in 2 L sterile conical flasks and mixed with 2 L of ethyl acetate. The mixture was vigorously vortexes for 1 h and allowed to stand for seven days at ambient temperature. The flask was monitored by vortexing 1 min every day for three times. The supernatant was separated by centrifugation at 1,000 g for 10 min and filtered through a No-2 filter paper. The filtrate was concentrated under reduced pressure at 40°C and further dried at room temperature until dryness. The crude extract, 20.4 g was packed in the column chromatography over silica gel and eluted with ethyl acetate (100%) followed by the combination of ethyl acetate: methanol by different concentration. The fractions were collected and checked for similar profile by thin layer chromatography (TLC) and then the fractions showing similar profiles were pooled. The solvent phase was concentrated using a vacuum at 35°C to obtain the crude extract for the antimicrobial bioassay. The pure compound was subjected to spectroscopic analysis by ^1^H and ^13^C nuclear magnetic resonance (NMR). The electrospray ionization mass spectra and infrared spectrum were also recorded.

### Antimicrobial activity

Antibacterial activity was carried out using a disc-diffusion method. The test cultures were swabbed on the top of the solidified media and allowed to dry for 10 min. The tests were conducted at different concentrations of the fractions. The loaded discs were placed on the surface of the medium and left for 30 min at room temperature for compound diffusion. Negative control was prepared using respective solvent. The plates were incubated for 17 h at 37°C for bacteria and 48 h at 30°C for fungi. Zones of inhibition were recorded in millimeters and the experiment was conducted in triplicates.

### Minimum inhibitory concentration (MIC)

The minimum inhibitory concentration was performed according to the standard reference method [9]. The compound was dissolved in water together with 2% dimethyl sulfoxide (DMSO). The initial test concentration (0.5 mg mL^−1^) was serially diluted twofold. Each well was inoculated with 5 μL of suspension containing 10^8^ CFU mL^−1^ of bacteria and 10^4^ spore mL^−1^ of fungi, respectively. For bacteria, the plates were incubated for 24 h at 37°C, whereas for fungi, the plates were incubated for 24, 48 or 72 h at 30°C. 5 μL of tested broth was placed on the sterile MHA plates for bacteria and incubated at respective temperature. The MIC for bacteria was determined as the lowest concentration of the compound inhibiting the visual growth of the test cultures on the agar plate. For, fungi, the MIC were calculated after an incubation time with no visible growth. The experiment was conducted in triplicates.

### Anti-biofilm activity of the compound

The anti-biofilm activity of the compound was studied at a concentration of 2–10 μg mL^−1^ against *E. coli*, *S. typhii* and *P. aeroginosa* respectively. Briefly, active test strains were grown in trypticase soy broth (TSB) and were added into the wells in triplicate, and sterile TSB was added as control to ensure that sterility of the medium throughout the experiment. The plates were aseptically sealed and incubated at 37°C for 8 h without shaking to allow cell attachment and biofilm development. After incubation, biofilm was stained with 0.4% crystal violet. The biofilm inhibitory concentration (BIC) was determined as the lowest concentration that produced visible disruption in biofilm formation and significant reduction in the readings when compared with the control wells at OD570_nm_, and the results expressed as percentage inhibition.

$$ \mathrm{Percentage}\ \left(\%\right)\ \mathrm{inhibition}=\frac{\mathrm{Absorbance}\ \mathrm{of}\ \mathrm{negative}\ \mathrm{control} - \mathrm{Absorbance}\ \mathrm{of}\ \mathrm{test}}{\mathrm{Absorbance}\ \mathrm{of}\ \mathrm{negative}\ \mathrm{control}}\times 100 $$

### *In Vitro* cytotoxicity assay (MTT assay)

The anticancer activity of the compound on HEP2 and Vero cell lines were determined by the MTT assay. Briefly, cells (1 × 10^5^/well) were plated in 0.2 mL of minimal essential media in 96-well plates and cultivated by incubating at 5% CO_2_ incubator for 72 h. Then, various concentrations of the compound in a 0.1% DMSO were added into the cells and incubated for 24 h. After incubation, the cells were removed by solution contained with phosphate-buffered saline (pH 7.4), 0.5% 3-(4,5-dimethyl-2-thiazolyl)-2,5-diphenyl--tetrazolium bromide (MTT) in phosphate- buffered saline. Viable cells were determined by measuring the absorbance at 540_nm_. Concentration required for a 50% inhibition of viability (IC_50_) was determined graphically. The effect of the compound on the proliferation of HEP2 and Vero cells was expressed as the percentage cell viability, using the following formula:

$$ \mathrm{Percentage}\ \left(\%\right)\ \mathrm{cell}\ \mathrm{viability} = {\mathrm{A}}_{540}\mathrm{of}\ \mathrm{treated}\ \mathrm{cell}\mathrm{s}/{\mathrm{A}}_{540}\mathrm{of}\ \mathrm{control}\ \mathrm{cell}\mathrm{s} \times 100 $$

### Antifeedant and Larvicidal activity of the compound

Larvae of *Helicoverpa armigera* and *Spodoptera litura* was collected from the field of Thuckalay, Kanyakumari district, Tamil Nadu, India, and cultured at room temperature (30°C) in the insectary and allowed to multiply. The larvae were fed with cotton and castor leaves soaked with 10% honey solution (Dabur Honey, India) mixed with a 50 μL of multi-vitamins (Hi-Media, Mumbai) was provided for adult feeding to increase the fecundity. The laboratory reared third instar larvae were used for the present investigation. Antifeedant activity of the compound was studied using leaf disc no choice method [10]. Briefly, fresh young cotton (*H. arigera*) and castor (*S. litura*) leaves were collected and cleaned thoroughly with water to remove the dust and other particles and the leaf discs of 4 cm in diameter were punched using a cork borer and were dipped in 0.5, 1.0, 2.5 and 5.0% concentrations of the compound individually. The leaf discs treated with water was used as negative control and azadirachtin (40.86% purity, obtained from EID-Parry, India Ltd., Chennai) was used as positive control. In each petri dish (1.5 × 9 cm), wet filter paper was placed to avoid the early drying of the leaf discs and one-third instar larva was introduced into each Petri dish. Progressive consumption of leaf area by the treated and control larvae after 24 h was recorded using the leaf area meter. Leaf area, eaten by larvae in treatment, was corrected from the negative control. Each concentration was maintained as five replicates with 10 larvae per replicate (total, N = 50). The experiment was performed in laboratory conditions (30 ± 2°C) with 14:10 photoperiod and 75 ± 5% relative humidity. Larvicidal activity was studied using leaf disc no-choice method by following Arasu et al. [11].

### Microorganisms

Bacteria: *Bacillus subtilis* (MTCC 441), *Enterococcus faecalis* (ATCC 29212), *Staphylococcus aureus* (ATCC 25923), *Staphylococcus epidermidis* (MTCC 3615), *Escherichia coli* (ATCC 25922), *Klebsiella pneumoniae* (ATCC 15380), *Pseudomonas aeruginosa,* (ATCC 27853), *Proteus mirabilis, Salmonella typhi* and fungi: *Aspergillus clavatus* (KACC 40071), *A. fumigates* (KACC 40080), *A. niger* (KACC 40280), *A. oryzae* (KACC 44823), *A. pullulans* (KACC 41291), *Botrytis cinerea* (KACC 40573), *B. elliptica* (KACC 43461), *Candida albicans* (KACC 30003), *C. acuminatus* (KACC 49383), *C. lunata* (KACC 40392), *Epidermophyton floccosum* (KACC 44918), *Fusarium culmorum* (KACC 42099), *F. oxysporum* (KACC 40051), *F. solani* (KACC 40384), *Gibberella moniliformis* (KACC 44022), *H. grisea* (KACC 40860), *Penicillium chrysogenum* (KACC 40399), *P. roqueforti* (KACC 41354), *Scytalidium lignicola* (KACC 41228), *S. vaccinii* (KACC 43121), *S. brevicaulis* (KACC 40273), *S. fusca* (KACC 41367), *Trichophyton mentagrophytes* (KACC 45479) and *T. roseum* (KACC 40956) were used for the experiment.

## Results

### Extraction and antimicrobial activity

**Figure 1:**
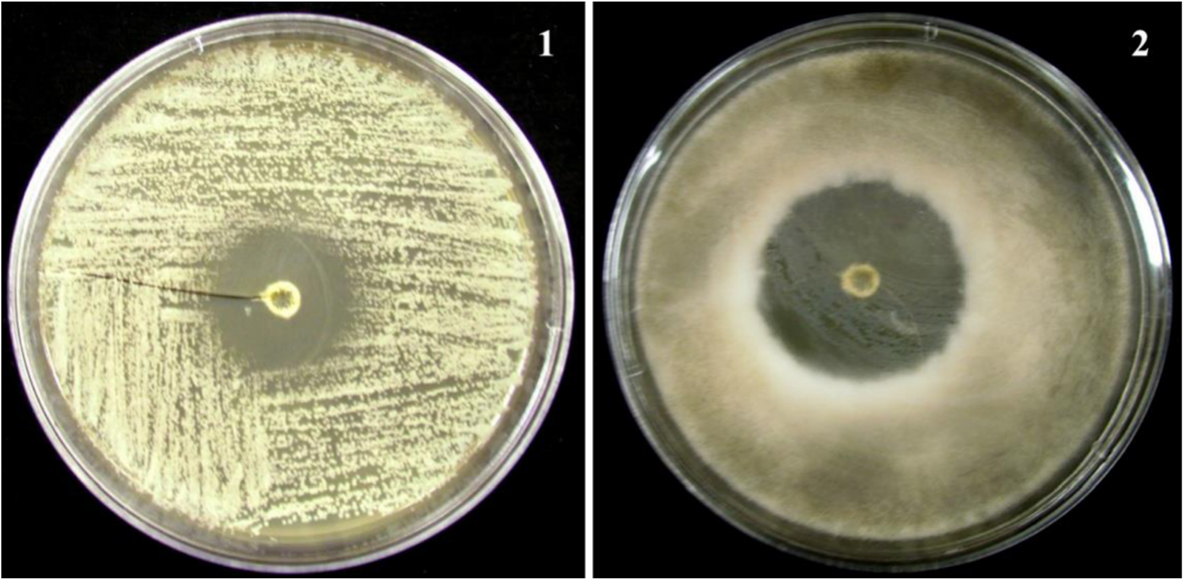
**Antimicrobial activity of the compound against microorganisms.** **1**., Activity against *Candida albicans.* **2**., Activity against *Aspergillus niger.*

**Figure 2:**
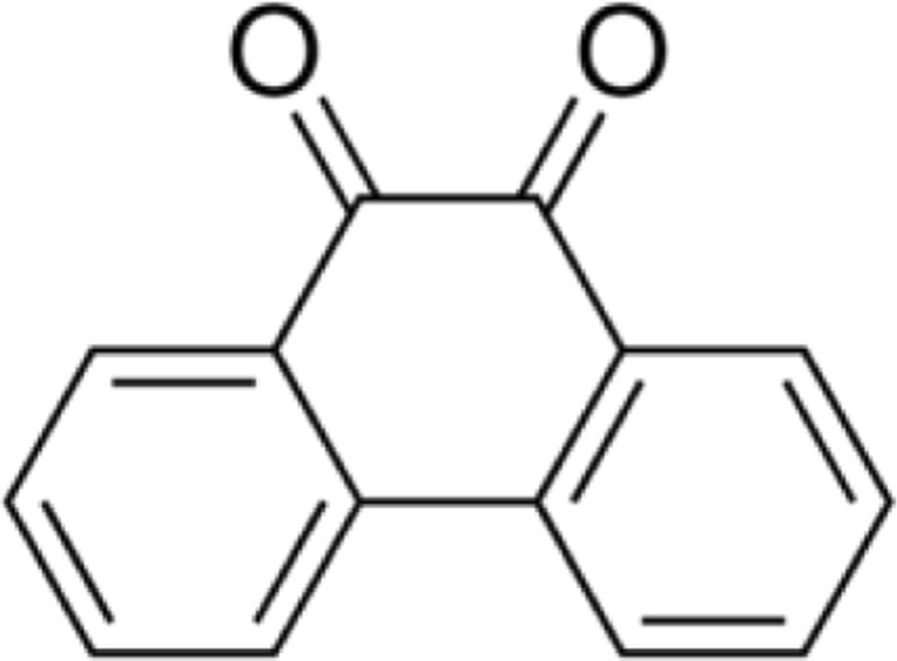
**Structure of quinone isolated from** ***Aegle marmelos*** **(Linn.) Correa.**

The antimicrobial activity of the ethyl acetate extracts against various Gram positive and Gram negative bacteria are summarized in Table 1. Primary screening by disc diffusion method showed zone of inhibition at 250 μg mL^−1^ for *B. subtilis*, whereas the other tested Gram positive bacteria such as *S. aureus*, *S. epidermidis* and *E. faecalis* revealed activity in 500 or 1000 μg mL^−1^ concentrations (Figure 1). All the tested Gram negative bacteria exhibited activity at 62.5 μg mL^−1^ except *P. mirabilis*. Primary screening results confirmed that the ethyl acetate extract could be promising to identify the novel compounds. Therefore, to obtain the pure compound, the crude ethyl acetate extract was re-extracted with different proportions of ethyl acetate and methanol and fractioned on silica gel. Twenty two fractions were pooled on the basis of thin layer chromatography (TLC) plate profile, concentrated and re-checked for their antimicrobial activity. The promising fractions against bacteria were chosen for further purification by re-extraction using a higher proportion of methanol and lower proportion of ethyl acetate and the purity by TLC. The homogeneity of the selected fractions was further confirmed by HPLC in which the metabolite was eluted from the column as a single peak. The IR spectrum of purified fraction exhibited strong absorption bands at 3100, 3060, 1600, 1590, 1450, 1430, 1280, 800 and 716 cm^−1^ which indicated hydroxyl, carbonyl, aromatic group and double bonds respectively; it agreed with the functional groups such as aromatic and hydroxyl stretching of quinone metabolite (Figure 2). ^1^H NMR (500 MHz) spectrum exhibited signals at 8.3, 8.2, 7.74, 7.7 and the ^13^C NMR spectrum showed signals at 180, 135.8, 135.5, 131, 129 and 124. These signals confirmed the presence of the ketone group in the structure of the metabolite. The MS spectrum showed major fragmentation at 208. The above spectral data guided us to draw the structure of the metabolite, and the chemical name of the metabolite was phenanthrenequinone. The molecular formula of the metabolite was C_14_H_8_O_2_.

**Table 1:** **Antibacterial activities of the ethyl acetate extract from** ***Aegle marmelos*** **(Linn.) Correa**

**Microorganism**	**Zone of inhibition (mm)**					
	**1000 μg**	**500 μg**	**250 μg**	**125 μg**	**62.5 μg**	**DMSO**
**Gram positive**						
*Bacillus subtilis*	20	18	12	NA	NA	NA
*Staphylococcus epidermidis*	15	10	NA	NA	NA	NA
*Enterococcus faecalis*	10	NA	NA	NA	NA	NA
**Gram negative**						
*Escherichia coli*	25	21	13	7	NA	NA
*Klebsiella pneumoniae*	15	12	10	7	NA	NA
*Psedomonas aeroginosa*	21	20	12	10	7	NA
*Proteus mirabilis*	NA	NA	NA	NA	NA	NA
*Salmonella typhi*	22	20	15	9	7	NA

Antimicrobial activities were assessed by incubating the strains at 37°C for 17 h. NA; No activity, Results were analyzed in triplicates.

### Antibacterial activity

The antibacterial profiles in terms of MIC of the isolated compound are shown in Table 2. The compound significantly suppresses the growth of the pathogens confirming the antimicrobial property. The organisms *E. coli*, *P. aeroginosa* and *S. typhi* were found to be more susceptible to the compound with MIC values of 25 μg mL^−1^. It showed MIC of 100 μg mL^−1^ towards Gram positive bacteria *B. subtilis* and *S. epidermidis,* whereas *S. aureus* and *E. faecalis* exhibited 75 and 50 μg mL^−1^ respectively. Among the Gram negative organisms, *P. mirabilis* and *K. pneumoniae* were moderately susceptible and showed higher MIC values (≥50 μg mL^−1^). The MIC values of the compound were marginally same as the standard broad spectrum antibiotic, streptomycin.

**Table 2:** **Minimum inhibitory concentration of the compound against bacteria**

**Microorganism**	**Minimum inhibitory concentration (MIC) (μg mL** ^**−1**^ **)**	
	**Compound**	**Streptomycin sulfate**
**Gram positive**		
*Bacillus subtilis*	100	2.5
*Staphylococcus aureus*	75	37.5
*Staphylococcus epidermidis*	100	10
*Enterococcus faecalis*	50	25
**Gram negative**		
*Escherichia coli*	25	25
*Klesiella pneumoniae*	50	25
*Pseudomonas aeroginosa*	25	50
*Proteus mirabilis*	100	50
*Salmonella typhi*	25	37.6

Minimum inhibitory concentration of the compounds were assessed by incubating the strains at 37°C for 17 h. Results were analyzed in triplicates.

### Antifungal activity

The antifungal activities of the isolated compound are shown in Table 3. The compound was effective against filamentous fungi such as *A. clavatus*, *A. niger*, *C. lunata*, *G. moniliformis*, and *P. roqueforti* with a MIC value of 20 μg mL^−1^. It showed significant antifungal activity against dermatphytes such as *S. fusca* (MIC: 80 μg mL^−1^), *T. roseum* (MIC: 100 μg mL^−1^), and *T. mentagrophytes*, *T. viridiae* (MIC: 125 μg mL^−1^). The MIC value more than 100 μg mL^−1^ were observed against organisms like *C. acuminatus*, *H. grisea*, *P. chrysogenum*, *S. vaccinii* and *S. brevicaulis*, while moderate MIC values (40 μg mL^−1^) observed towards *A. fumigates*, *A. flavus*, *A. oryzae*, *E. floccosum*, *F. culmorum* and *S. lignicola* respectively. The MIC values of the compound (60 μg mL^−1^) against *C. albicans* were slightly lesser than the standard antibiotics, ketaconazole. The standard antifungal agent exhibited MIC values ranged from 25–100 μg mL^−1^) respectively.

**Table 3:** **Minimum inhibitory concentration of the compound against fungi**

**Microorganism**	**Minimum inhibitory concentration (MIC) (μg mL** ^**−1**^ **)**	
	**Compound**	**Standard**
*A. clavatus*	20	50
*A. furnigates*	40	50
*A. flavus*	40	25
*A. niger*	20	25
*A. oryzae*	40	50
*A. pullulans*	80	37.5
*B. cinerea*	100	50
*B. elliptica*	80	50
*C. albicans*	60	100
*C. acumninatus*	100	37.5
*C. lunata*	20	50
*E. floccosum*	40	37.5
*F. culmorum*	40	25
*F. oxysporum*	20	25
*F. solani*	60	75
*G. moniliformis*	20	100
*H. grisea*	100	50
*P. chrysogenum*	100	25
*P. notatum*	60	25
*P. roqueforti*	20	25
*S. lignicola*	40	50
*S. vaccinii*	100	25
*S. brevicaulis*	100	25
*S. fusca*	80	50
*T. mentagrophytes*	125	50
*T. roseum*	100	50
*T. viridiae*	125	50

Minimum Inhibitory concentration of the compounds were assessed by incubating the strains at 37°C for 24, 48 or 72 h at 30°C. Results were analyzed in triplicates.

### Anti-biofilm activity

**Figure 3:**
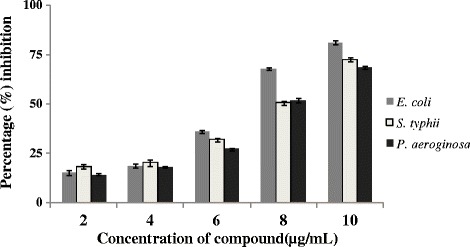
**Antibiofilm activity of the compound against microorganisms.**

*In vitro* anti biofilm activity of the compound against *E. coli*, *S. typhii* and *P. aeroginosa* were monitored by assaying crystal violet dye and the reduction in cell attachment was measured spectrophotometerically. Results indicated that 8 μg mL^−1^ concentration reduced 68%, 50.6% and 52% of 50% of *E. coli*, *S. typhii* and *P. aeroginosa* cell attachment (Figure 3). Maximum reduction in cell attached observed in 10 μg mL^−1^ concentrations. The compound exhibited a significant dose dependent inhibition of biofilm formation, with the highest activity (81.33%, 72.66% and 68.77%) at 10 μg mL^−1^ concentrations.

### Cytotoxicity

**Figure 4:**
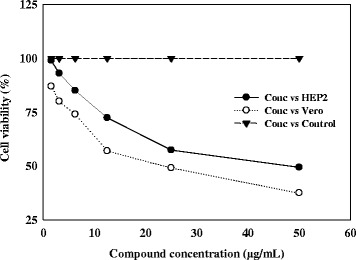
**Cytotoxic effect of compound on Vero and HEP2 cell lines as determined by MTT assay.**

**Figure 5:**
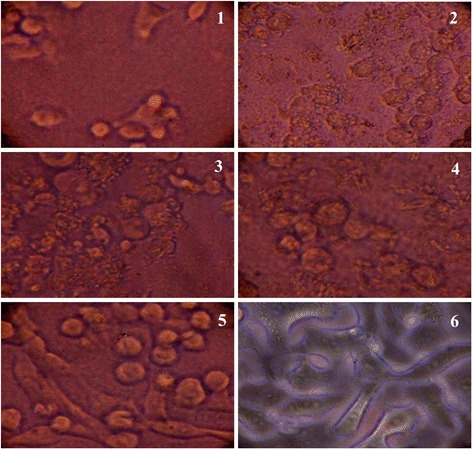
**Cytotoxic effect of compound on HEP2 cell line.** **1**; 25 μg ML^−1^, **2**; 12.5 μg ML^−1^, **3**; 6.25 μg ML^−1^, **4**; 3.12 μg ML^−1^, **5**; 1.26 μg ML^−1^ concentration of the compounds; **6**; without treatment.

The cytotoxic effect of different concentrations of the compound to a normal Vero cell lines and HEP2 cell lines were evaluated by MTT assay in 96-well plates. Different concentrations (1.56–50 μg) of the compound on the tested cells exhibited concentration and time dependent inhibition (Figures 4 and 5). IC_50_ values were calculated as 25 and 10 μg mL^−1^ for Vero and HEP2 cell lines, respectively. The results indicated that the compound was comparatively nontoxic to normal cells.

### Antifeedant and larvicidal activity

Antifeedant activity of the compound against *H. armigera* and *S. litura* is presented in Table 4. The metabolite showed strong antifeedant activity of 73% and 78.12% against *H. armigera* and *S. litura*, respectively at 1000 ppm concentration and 62.76% and 65.72% at 500 ppm concentration respectively. The metabolite documented 63.6% and 71.8% larvicidal activities against *H. armigera* and *S. litura*, respectively at 1000 ppm and the activity was slightly near to the concentration of the standard antifeedant compound. The LC_50_ value was 786.16 and 696.37 ppm for *H. armigera* and *S. litura*, respectively.

**Table 4:** **Antifeedant and Larvicidal activity of the metabolite against** ***H. armigera*** **and** ***S. litura***

**Concentration (ppm)**	**Antifeedant activity (%)**		**Larvicidal (%)**	
	***Helicoverpa armigera***	***Spodoptera litura***	***Helicoverpa armigera***	***Spodoptera litura***
*Compound*				
125	32.44 ± 2.21	40.52 ± 1.32	23.8 ± 2.16	32.0 ± 1.41
250	41.92 ± 1.49	47.64 ± 5.12	34.4 ± 0.89	40.6 ± 0.54
500	62.76 ± 1.39	65.72 ± 2.01	54.2 ± 0.83	55.4 ± 1.14
1000	73.00 ± 3.82	78.12 ± 1.92	63.6 ± 1.94	71.8 ± 2.38
*Azadirachtin*				
125	60.36 ± 1.31	61.32 ± 1.24	45.2 ± 1.48	49.4 ± 2.5
250	66.68 ± 1.31	71.48 ± 2.29	62.6 ± 1.51	67.4 ± 1.81
500	77 ± 1.73	82.72 ± 3.31	98.8 ± 1.3	97.6 ± 1.31
1000	91.88 ± 2.22	86.56 ± 2.91	100 ± 00	100 ± 00
*Control*	5.3 ± 1.72	2.24 ± 1.3		

Data are mean ± SD.

## Discussion

In the present study, a thorough analysis guided to identify novel metabolite from medicinal plants and its broad applications towards pathogenic bacteria, fungi, insects and cancer cells. The finding of new bioactive compounds from medicinal plants is a never ending process to meet the everlasting demand for novel bio-molecules with antimicrobial properties in order to combat human and plant pathogens. It is more important to screen traditional medicinal plants for bioactive compounds because they are the essential sources of potent molecules. Medicinal plants spread in Kanyakumari district is known for chemical and biological diversity [12-14]. Biological applications of medicinal plants from Kanyakumari district are well documented; yet the identification of active compounds of these plants which represents the indigenous traditional usages remains unclear. *Aegle marmelos* (Linn.) Correa was selected in this study based on its traditional use for treating fever, diarrhea, stomachache, gastric troubles dysentery and inflammation in human and veterinary medicine. Its leaf is used to cure optalmia and venereal diseases, whereas the dried fruits used in the treatment of diarrohea and dysentery. However, the antimicrobial properties of this plant against pathogenic bacteria and spoilage fungi were rarely studied. The bacterial strains used in this study are those recommended for antibacterial activity testing by the United States National Committee for Clinical Laboratory Standards and the fungal pathogens used in the screening are important opportunistic pathogens for human and agriculture. The ethyl acetate extract recovered from the plant exhibited significant antimicrobial activity against Gram positive and Gram negative pathogens, therefore, purification of the compound was carried out in a silica column, and the compound was chemically characterized by ^1^H NMR and ^13^C NMR. The identified metabolite was identified as quinone in nature.

*In vitro* antimicrobial assays against standard strains have demonstrated various degrees of growth inhibition. The compound was active against Gram positive and Gram negative bacteria, filamentous and dermatophytic fungi. The results confirmed that the antimicrobial activity of the compound was highly active against Gram negative bacteria compared to Gram positive. The antimicrobial activity of *Aegle marmelos* (Linn.) has not been previously described, but other members of the *Rutaceae* family demonstrated antibacterial and antifungal activity [15,16].

The antibiofilm potential of the compound against *E. coli*, *S. typhii* and *P. aeroginosa* is a promising tool for reducing microbial colonization on the surfaces and the epithelial mucosa leads to infections or by interrupting the release of the adhesion compound lipoteichoic acid from the streptococcal cell surface. Many plant extracts were evaluated for antibiofilm activity against different bacteria with higher concentration of the compound whereas; in this study the compound exhibited antimicrobial activity at lower concentrations in the range of 20-125 μg mL^−1^ [17]. Therefore, its antibiofilm activity was evaluated in lower concentration. It is predicted that the isolated compound has produced an unfavourable environmental conditions that promotes detachment of the bacteria, thereby reducing the surface adhesion, the antimicrobial susceptibility of the compound toward drug resistance strains, the compound may prevent the generation of the extracellular polysaccharides such lipopolysaccharides, fimbriae and pilli, neutralizing the negative charge of the extra cellular poly saccharides in the bacterial cells by donating the positive charge of the compound and inhibiting the efflux pump mechanism of the drug resistance pathogens [18,19].

The compound exhibited potent inhibitory activity against HEP2 and Vero cell line with an IC_50_ value of 25 μg mL^−1^. It did not show toxicity to the normal cells therefore it could be used in the treatment of cancer. Reports claimed that plant metabolites belonged to quinone classification exhibited wide range of biological activities, including antioxidant, anti-proliferation, anti-inflammation and anticancer activities [20,21]. The assessment of cytotoxicity is very important and a crucial step in the development of new therapeutic drugs for clinical application.

In addition, the identified compound showed more than 73% and 78% antifeedant activity against *H. armigera* and *S. litura*. Similarly, many plant derived metabolites such as 6-(4,7-hydroxyhyptyl) quinine, 2E)-1-(2-hydroxyphenyl) pent-2- en-1-one, 1-[(3-hydroxy-5,5-dimethylcyclohex- 3-en-1-yl)oxy]hexane-3-one, rhein, momordicin I, momordicin II, ononitol monohydrate, 2,5-diacetoxy-2-enzyl-4,4,6,6-tetramethyl-1,3- cyclohexanedi one revealed antifeedant and larvicidal activity against *H. armigera* and *S. litura* [22-27]. The isolated compound in this study moderately inhibited the growth of *H. armigera* and *S. litura* as compared to the standard compound therefore; it could be alternatively used to protect the crops from insects.

## Conclusions

In conclusion, spectroscopic and chromatographic analytical methods guided to identify the novel phenanthrenequinone compound from the ethyl acetate extract of *Aegle marmelos* (Linn.). The compound had good antibacterial activity against Gram positive and negative bacteria. It inhibited the growth of spoilage fungi in the concentration of 20 -125 μg mL^−1^ . It also had a concentration dependent antibiofilm activity against *E. coli*, *S. typhii* and *P. aeroginosa* respectively. Besides that, the compound exhibited cytotoxic activity against carcinoma cell lines *in vitro*. The potent antifeedant and larvicidal activities of the compound against *H. armigera* and *S. litura* could be used for the preparation of new pesticide formulations for the control of agricultural pest. The wide application of this plant provides a scientific basis for the use of the plants in traditional medicines to kill the pathogenic microorganisms and to control the agriculture pests which added advantage of the plants.
